# Characterization of the Notch pathway in nasal polyps of patients with chronic rhinosinusitis: A pilot study

**DOI:** 10.14814/phy2.15403

**Published:** 2022-08-27

**Authors:** Giorgio Aquila, Alessandra Alaimo, Luisa Marracino, Valeria Martino, Francesca Camponogara, Francesco Vieceli Dalla Sega, Francesca Fortini, Antonio Pannuti, Claudia Zanotti, Nicola Malagutti, Stefano Pelucchi, Paola Rizzo

**Affiliations:** ^1^ Department of Medical Sciences University of Ferrara Ferrara Italy; ^2^ Department of Ear, Nose and Throat University Hospital of Ferrara Ferrara Italy; ^3^ Department of Translational Medicine and Laboratory for Technologies of Advanced Therapies (LTTA) University of Ferrara Ferrara Italy; ^4^ University of Hawaii Cancer Center, University of Hawaii Honolulu Hawaii USA; ^5^ Department of Neuroscience DNS, Section of Otolaryngology University of Padova Padova Italy

**Keywords:** eosinophils, glucocorticoids, nasal polyps, notch pathway, rhinosinusitis, translational medicine

## Abstract

Chronic rhinosinusitis with nasal polyps is a widespread pathology characterized by persistent inflammation of nasal and paranasal mucosa. Although it represents one of the most frequent diseases of the nasal cavities, its etiology is still not completely elucidated. There is evidence suggesting that the Notch signaling, a highly conserved intercellular pathway known to regulate many cellular processes, including inflammation, is implicated in nasal polyps formation. The purpose of this study was to investigate the expression of genes of the Notch pathway in nasal polyps from patients with chronic rhinosinusitis. Nasal polyps and adjacent mucosa tissue were obtained from 10 patients. RNA was analyzed by quantitative reverse transcriptase‐polymerase chain reaction for the expression level of (1) Notch pathway components such as receptors (NOTCH1‐4), ligands (DLL4, JAGGED‐1), and target genes (HEY1, 2, and HES1) and (2) genes providing information on the pathogenesis of polyposis (C‐MYC and SCGB1A1) and on eosinophils content (CCL26, IL5, and SAA2). We report a Notch‐driven gene expression pattern in nasal polyps which correlates with the expression of genes highly expressed in eosinophils, whose presence is an important parameter to define the pathophysiologic diversity characterizing nasal polyps. Taken together, our results suggest a role for Notch signaling in the pathophysiology of polyposis. Further studies are needed to elucidate the role of Notch in nasal polyps formation and to establish whether it could represent a novel therapeutic target for this pathology.

## Graphical abstract

Activation of the Notch pathway in nasal polyps could be dependent by the ligand JAGGED‐1. NECRSwNP was characterized by higher level of Notch signaling. Notch inhibitors, in combination with glucocorticoids, could be a possible therapeutic treatment for nasal polyps.

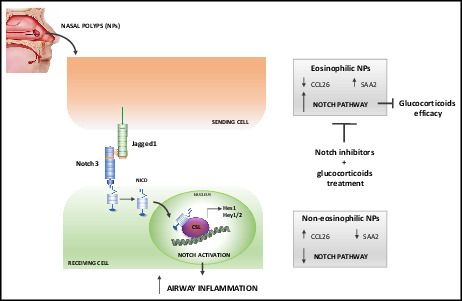

## 1 INTRODUCTION

Chronic rhinosinusitis (CRS) is a heterogeneous group of sinus diseases characterized by persistent inflammation of the sinonasal mucosa with a significant impact on patient quality of life (Hopkins, 2019; Kucuksezer et al., 2018; Schleimer, 2017). CRS is generally divided into two types, according to the absence or presence of nasal polyps (NPs): CRS without nasal polyps (CRSsNPs) and CRS with nasal polyps (CRSwNPs) (Chaaban et al., 2013). NPs are chronic recurrent inflammatory processes characterized by edema and hyperplasia of the mucosa of the paranasal sinuses, with a prevalence of 4% in the general population and a peak onset between the fourth and sixth decade (Newton & Ah‐See, 2008). CRSwNPs can present either NPs characterized by pronounced eosinophil infiltration (ECRSwNP), which are typically associated with non‐allergic late‐onset asthma but accentuated by co‐existing allergic rhinitis (Rosati & Peters, 2016), or NPs associated with neutrophilic inflammation or non‐eosinophilic (NECRSwNP) (Cao et al., 2009; Kato, 2015; Meltzer et al., 2004). ECRSwNP patients present more severe symptoms compared to NECRSwNP patients and are associated with polyposis relapse (Nakayama et al., 2011). ECRSwNP and NECRSwNP display distinct gene expression (Wang, Zhang, et al., 2016) and immunological/inflammatory profiles (Wang, Zhang, et al., 2016); it has been suggested that T helper (Th) 2 is the predominant T‐cell subset in ECRSwNPs, while Th17 is predominant in NECRSwNPs (Sun et al., 2017), although other studies seem to indicate a more complex heterogeneity (Tomassen et al., 2016; Wang, Zhang, et al., 2016; Workman et al., 2018). In fact, previous studies showed an involvement of systemic human leukocyte antigen G (HLA‐G) system alterations among CRSwNPs since these patients were unable to express HLA‐G molecules in peripheral blood monocytes even in presence of interleukin (*IL)‐10* (HLA‐G main inducer) (Malagutti et al., 2008). Mutations of *IL‐10* gene have been excluded in CRSwNPs (Malagutti et al., 2015), whereas a possible role of Human Papilloma Virus (HPV) 11 in recurrences of massive nasal polyposis has been shown (Rizzo et al., 2014).

The etiology of nasal polyposis is complex and not completely understood and, thus, it is not known why some patients with CRS develop NPs. There is evidence suggesting an involvement of the Notch signaling in NPs. The Notch signaling controls multiple biological processes such as cell proliferation, survival, and differentiation (Guruharsha et al., 2012; Rodriguez‐Vita et al., 2017), and, when dysregulated, it plays a prominent role in many types of malignancies (Espinoza & Miele, 2013a) and in inflammatory diseases (Fazio & Ricciardiello, 2016; Quillard & Charreau, 2013; Rizzo et al., 2013), including local airway inflammation (Huang et al., 2017; Tindemans et al., 2017). The Notch signaling is initiated by the interaction between the Notch receptors (Notch1‐4) and their ligands of the Delta‐like families (Delta‐like ligand [DLL] 1, 3, and 4) or Jagged families (Jagged‐1 and ‐2). The binding of the ligand to the receptor leads to two proteolytic cuts, the last cut being mediated by the γ‐secretase, that releases the Notch intracellular domain (NICD), the active form of the receptor. NICD translocates into the nucleus and binds the transcription factor CSL (CBF‐1, Suppressor of Hairless and Lag‐1) thus promoting the transcription of Notch target genes. The best‐characterized Notch target genes belong to the Hairy and Enhancer of Split (*HES*) and Hairy and Enhancer of Split with YRPW motif (*HEY*) gene families (Kovall et al., 2017; Malagutti et al., 2008).

Notch is an important modulator of cells effectors of acquired and innate immunity (Radtke et al., 2010; Shang et al., 2016), involved in NPs formation (Kovall et al., 2017; Lane, 2009; Tan et al., 2017). In a mouse model of chronic allergic rhinitis, treatment with DAPT (N‐[N‐(3,5‐Difluorophenacetyl)‐L‐alanyl]‐S‐phenylglycine t‐butyl ester), a γ‐secretase inhibitor, alleviated upper airway inflammation by suppressing Th2 cytokine levels, reducing eosinophils infiltration and goblet cells metaplasia (Shi et al., 2017). Furthermore, differential expression of NOTCH1 has been observed between NPs and sinonasal inverted papillomas (Karagianni et al., 2018). Notch regulates the expression of endothelial nitric oxide synthase (eNOS) (Patenaude et al., 2014), clara (Club) cell 10 kDa protein (CC10) (Motooka et al., 2017), and transforming growth factor‐β (TGF‐β) (Kluppel & Wrana, 2005), all known to be differently regulated in NPs compared to normal mucosa (Coste et al., 1998; Fritz et al., 2003; Koennecke et al., 2018). Notch also regulates cell growth (Song & Lu, 2011), which has been shown to be deregulated in NPs (Fritz et al., 2003). A recent study has provided evidence of the involvement of Jagged‐1‐mediated Notch1 activation in CRSwNPs through the hyperexpression of IL‐33 (Chiappara et al., 2019). It is well known that the output of the Notch pathway is extremely context dependent (Espinoza & Miele, 2013b): Notch1‐4 receptors can activate distinct pathways, also acting in antagonism between each other (Benedito et al., 2009). Furthermore, the activation of Notch receptors by DLL4 can activate a different set of genes, compared to activation by Jagged‐1 (Aquila et al., 2017).

Based on this evidence, the aim of our study was to further investigate the involvement of Notch in the pathophysiology of NPs by determining the mRNA expression levels of genes related to Notch signaling in NPs of patients with CRS. We also determined the expression levels of genes regulated by Notch and known to influence the biology of NPs. Since hyperplasia of nasal epithelial cells plays a role in the formation of NPs (Hellquist, 1996), we analyzed the expression of *C‐MYC*, a Notch target gene regulating cell proliferation (Espinoza & Miele, 2013a), known to be expressed in NPs (Koennecke et al., 2017) and involved in response to glucocorticoids NP treatment (Lin et al., 2008). We then assessed the mRNA levels of *SCGB1A1* (uteroglobin, member of the secretoglobin family), a protein constitutively expressed in the epithelial cells of the nose and an important mediator of inflammatory and allergic responses (Liu et al., 2013; Singh & Katyal, 2000) known to be dysregulated in NPs (Liu et al., 2009; Lu et al., 2011) and regulated by Notch (Tsao et al., 2009; Xing et al., 2012). To investigate a possible association between Notch signaling and NP content of eosinophils, we determined the expression levels of the genes *CCL26* (c‐c motif chemokine ligand 26), *IL5* (Interleukin‐5), and *SAA2* (serum amyloid A2) mRNA. *CCL26* and *IL5*, which result highly expressed in ECRSwNP (Tian et al., 2019) (Bachert et al., 1997) (Lou et al., 2018; Yan et al., 2019), act as chemotactic agents for eosinophils (Brussino et al., 2018; Kramer & Rasp, 1999; Wang, Zhang, et al., 2016), whereas SAA2 is a protein secreted during the acute phase of inflammation which is 25‐fold more expressed in NECRSwNPs compared to ECRSwNPs (Wang, Gao, et al., 2016).

## 2 MATERIALS AND METHODS

### 2.1 Patients and samples

Ethics Authority (Comitato Etico Unico della Provincia di Ferrara, 355/2019/Oss/AOUFe) approved the study. All patients gave their written informed consent. Ten patients with CRS and NPs were enrolled (6 men, 4 women, with a mean (*SD*) age of 55.2 (11.35) years). The diagnosis was made by otolaryngologists of the ENT Department at the University Hospital of Ferrara following criteria defined in Chapter 1.2.2.1. of the European Position on Rhinosinusitis and Nasal Polyps (presence of inflammation of the nose and the paranasal sinuses characterized by either nasal blockage, obstruction, and congestion or nasal discharge; patients referred also facial pain or pressure with reduction or loss of smell; endoscopic signs of nasal polyps and typical CT mucosal changes related to common CRSwNP). The pathological examination was confirmed by endoscopy of both nostrils and computer tomography scan of the nose. All subjects had NPS score of 6 or higher. They were all primary cases were excluded: smokers, allergic patients, previous use of topic drugs, nasal polyps associated with odontogenic sinusitis, patients with important anatomical abnormalities such as concha bullosa or important septal deviations, immunodeficiency, cystic fibrosis, bronchiectasis, chronic obstructive pulmonary disease, diabetes mellitus, neoplasia, and oral steroid treatment. Details about the study population are shown in Table 1. All the subjects used oral or nasal corticosteroids before surgery. Given the fact that there are no much data in the literature on interindividual variability in the expression levels of the components of the Notch pathways in normal airways mucosa, we reasoned that the best control would be the normal tissue adjacent to the polyp (Fritz et al., 2003) and thus, for each patient, NPs and adjacent mucosa (AM) were analyzed. Samples were immediately preserved in RNA Later solution (Qiagen) and stored at −80°C.

**TABLE 1:** Characteristics of patients

Variables	Patients (*n* = 10)
Age mean (*SD*)	55.2 (11.35)
Male (%)	6 (60)
NPS score	5: NPS 6 3: NPS 7 2: NPS 8
Ex‐ Smokers (%)	3 (30)
Drug intolerances (%)	2 (20)
Other allergies (%)	6 (60)
Recurrences (%)	2 (20)
Glucocorticoid therapy (%)	10 (100)

Abbreviation: *SD*, standard deviation.

### 2.2 Quantitative RT‐PCR analysis

Total RNA was isolated from 10 to 20 mg of tissue by the RNeasy Fibrous Tissue Mini Kit (Qiagen) according to the manufacturer's protocol. RNA concentration and purity were determined by NanoDrop 2000 spectrophotometer (Thermo Fisher Scientific). Total RNA (500 ng) was reverse transcribed in a volume of 25 μl using 250 units of SuperScript III reverse transcriptase (Life Technologies) and 50 ng of random hexamers. Two microliters of the cDNA mixture was used for real‐time PCR experiments to measure the amount of *NOTCH1*, *NOTCH2*, *NOTCH3*, *NOTCH4*, *HES1*, *HEY1*, *HEY2*, *JAGGED‐1*, *DLL4*, *SCGB1A1*, *SAA2*, *C‐MYC*, *CCL26*, and *IL5* transcripts. *RPL13* and *GUSB* were used as internal reference genes. Real‐time PCR reactions were conducted on a 7500 Fast Real‐Time PCR System (Applied Biosystems, Life Technologies) using PerfeCta SYBR Green SuperMix with ROX kit (Quanta Biosciences) according to the manufacturer's protocol in a final volume of 23 μl. The sequences of primers used are shown in Supplementary Table S1. Primers were purchased from IDT. Differences in gene expression levels between NP and AM were determined by the 2^−ΔΔCt^ formula (Aquila et al., 2018). For two genes (*CCL26* and *SAA2*), the 2^−ΔΔCt^ values were calculated using either *RPL13* or *GUSB* as reference gene and no differences in quantification were observed (Supplementary Figure S1). Based on these data, for all the other genes we used *RPL13* as an internal reference gene. For correlation and clustering analyses of values obtained from NP and AM samples, ΔCt values (ΔCt = Ct target gene ‐ Ct *RPL13*) were used.

### 2.3 Statistical analysis

Normal distribution of the variables was verified with the D'Agostino–Pearson normality test and with the Shapiro–Wilk test (alpha = 0.05). Variables were presented as scatter plot with median. Two‐tailed Student's *t* test was used to test differences between the mean (NPs vs. AM sample) and a *p* < 0.05 was considered to be statistically significant. Hierarchical clustering was performed using the Genepattern engine (http://genepattern.broadinstitute.org) with pairwise average‐linkage as clustering method; column and row distance measures were calculated using Spearman's rank correlation. The gene expression correlation matrix was generated from ΔCt values for each target gene using the GraphPad Prism 6 software (https://www.graphpad.com/).

## 3 RESULTS

### 3.1 Differential expression of notch pathway components between nasal polyps and adjacent mucosa

**FIGURE 1:**
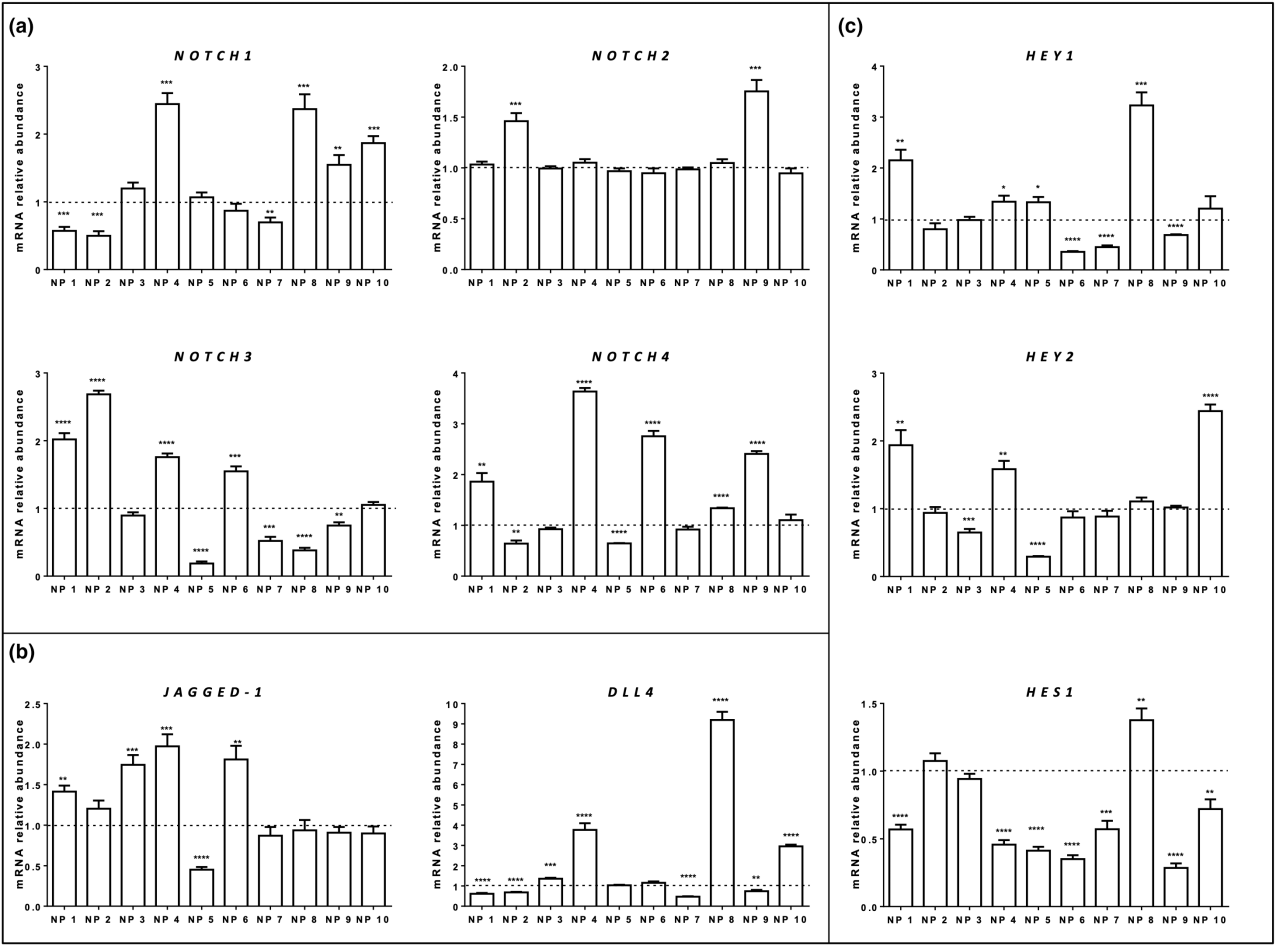
Expression of notch pathway components in nasal polyps in patients with chronic rhinosinusitis. Expression level of NOTCH pathway receptors (*NOTCH 1–4*; (a), ligands (*JAGGED‐1, DLL4*; (b) and target genes (*HEY1, 2* and *HES1*; (c) was assessed using qRT‐PCR analysis. Gene expression levels in nasal polyps (NP) were compared to the levels in adjacent mucosa (AM) using the 2^−∆∆Ct^ formula and RPL13A as reference genes. Results are expressed as mean ± *SEM* of at least three experiments. ****p < 0.0001, ***p < 0.001, **p < 0.01, and *p < 0.05, NP vs the corresponding AM samples. The dotted line represents the normalized expression levels in AM samples.

**FIGURE 2:**
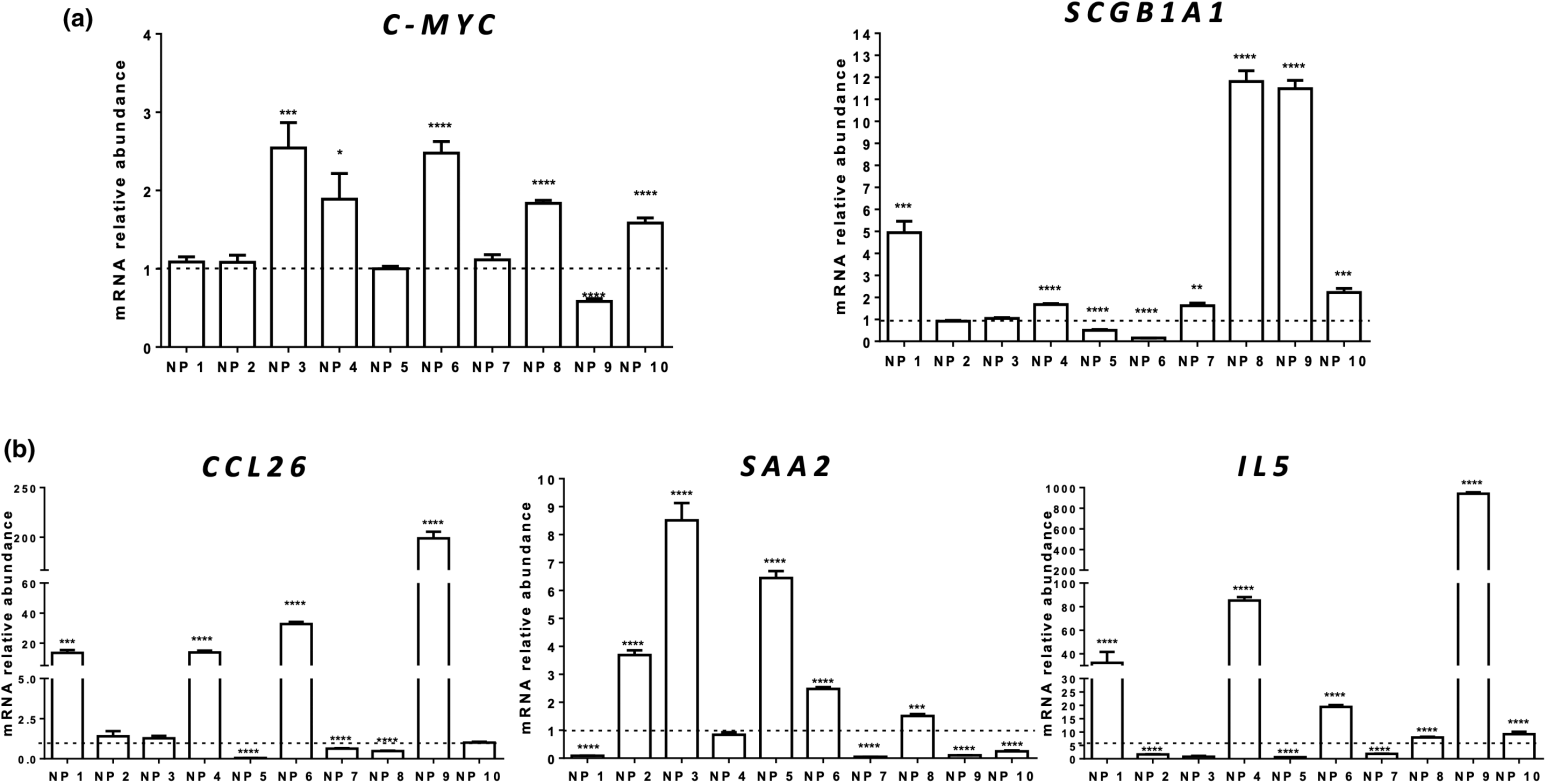
Expression of biological relevant genes in nasal polyps of patients with chronic rhinosinusitis. (a) *SCGB1A1*, *C‐MYC*, (b) *SAA2, CCL26*, and *IL5* genes expression was assessed using qRT‐PCR analysis. Gene expression levels in nasal polyps (NP) were compared to the levels in adjacent mucosa (AM) using the 2^−∆∆Ct^ method and RPL13A as reference gene. Results are expressed as mean ± *SEM* of at least three experiments. ****p < 0.0001, ***p < 0.001, **p < 0.01, and *p < 0.05, NP vs the corresponding AM samples. The dotted line represents the normalized expression levels in AM samples.

Quantitative RT‐PCR analyses showed that Notch receptors (*NOTCH1, 2, 3*, and *4*), ligands (*DLL4* and *JAGGED‐1*), and target genes (*HES1, HEY1*, and *HEY2*) were all expressed both in NP and AM samples and there were differences between NPs and AM in the expression levels of every Notch pathway component analyzed (Figure 1; Table 2). In particular, *NOTCH1* was upregulated (4/10) and downregulated (3/10) in NPs, compared to the corresponding AM, while in the remaining three patients there were no changes in *NOTCH1* expression between NP and AM (Figure 1a). *NOTCH2* was upregulated in NP of two of 10 patients, in the remaining patients there was no significant difference between NP and AM in *NOTCH2* mRNA levels (Figure 1a); *NOTCH3* was upregulated in NP of four of 10 patients, downregulated in NPs of other four patients and in the remaining two patients did not result differently expressed between NPs and AM (Figure 1a); *NOTCH4* was upregulated in NP of five patients, downregulated in NP of two patients and in the remaining three patients there was no difference between NP and AM (Figure 1a). *JAGGED‐1* mRNA was upregulated in pathological NP of four of 10 patients, downregulated in NP of only one patient and in the remaining five patients *JAGGED‐1* was not differently expressed between NP and AM (Figure 1b). *DLL4* was upregulated in NP of four of 10 patients compared with AM, downregulated in NP of four patients and in the other two patients there was no difference in its expression level between NP and AM (Figure 1b). Concerning the expression of Notch target genes, we found that *HEY1* was upregulated in NP from four of 10 patients and downregulated in NP of three of 10 patients, compared to the corresponding A,M whereas in the remaining three patients there was no difference in its expression level between NP and AM (Figure 1c); *HEY2* resulted upregulated in NPs of three of 10 patients, downregulated in NP of two patients and in the remaining five patients there was no difference between NP and AM (Figure 1c). *HES1* was upregulated in NP of only one patient, compared to AM, downregulated in seven of 10 patients and in the remaining two patients was not differently expressed between NP and AM (Figure 1c). *C‐MYC* resulted upregulated in NPs of five of 10 patients, downregulated in one patient and was not differently expressed, between NP and AM, in the remaining four patients (Figure 2a). We found that *SCGB1A1* was upregulated in NPs of six of 10 patients compared to AM, downregulated in NP of two patients and it was not differently expressed, between NP and AM, in the remaining two patients (Figure 2a). The expression of *SAA2*, in NP, was upregulated in five of 10 patients, downregulated in four patients and in the remaining patient it was not differently expressed (Figure 2b). *CCL26* resulted upregulated in NP of four of 10 patients compared to their corresponding AM, downregulated in NP of three patients and in three patients there is no difference between AM and NP group in *CCL26* expression levels (Figure 2b). *IL5* was upregulated in NP of six of 10 patients compared to their corresponding AM, downregulated in NP of three patients and it was not differently expressed between AM and NP group in one patient (Figure 2b).

**TABLE 2:** Schematic representation of changes in the expression levels of notch pathway components in nasal polyps (ID1‐10), compared to the adjacent mucosa

	*NOTCH1*	*NOTCH2*	*NOTCH3*	*NOTCH4*	*DLL4*	*JAGGED‐1*	*HEY1*	*HEY2*	*HES1*
ID 1	−^a^	/	++	+^c^	‐‐^b^	++^d^	++	++	−
ID 2	−	+	++	−	−	/	/	/	/
ID 3	/	/	/	/	+	++	/	−	/
ID 4	++	/	++	++	++	++	+	++	−
ID 5	/	/	−	−	/	−	+	−	−
ID 6	/	/	+	++	/	++	−	/	−
ID 7	−	/	−	/	−	/	−	/	−
ID 8	++	/	−	+	++	/	++	/	+
ID 9	+	++	−	++	−	/	−	/	−
ID 10	++	/	/	/	++	/	/	++	−

^a^ Fold‐change < 0.7;

^b^ Fold‐change < 0.5;

^c^ Fold‐change > 1.3;

^d^ Fold change > 1.5.

The expression levels for all the analyzed genes, except for *HES1*, were highly heterogeneous both in NP and AM and thus, no differences were observed in gene expression levels between the pool of NP and the pool of AM (Supplementary Figures S2 and S3). It is possible that these results are due to the spreading to the adjacent mucosa of biological processes ongoing in the polyps. This hypothesis is consistent with previous studies (Biggs et al., 2019; Hao et al., 2006). Given this fact, some AM could be unsuitable for a comparison with the matched NP. For this reason, we performed separate correlation and clustering analyses for mRNA levels in NP and AM.

### 3.2 Correlation analysis identifies co‐expression profiles of notch pathway genes specific to NP and AM biopsies

**FIGURE 3:**
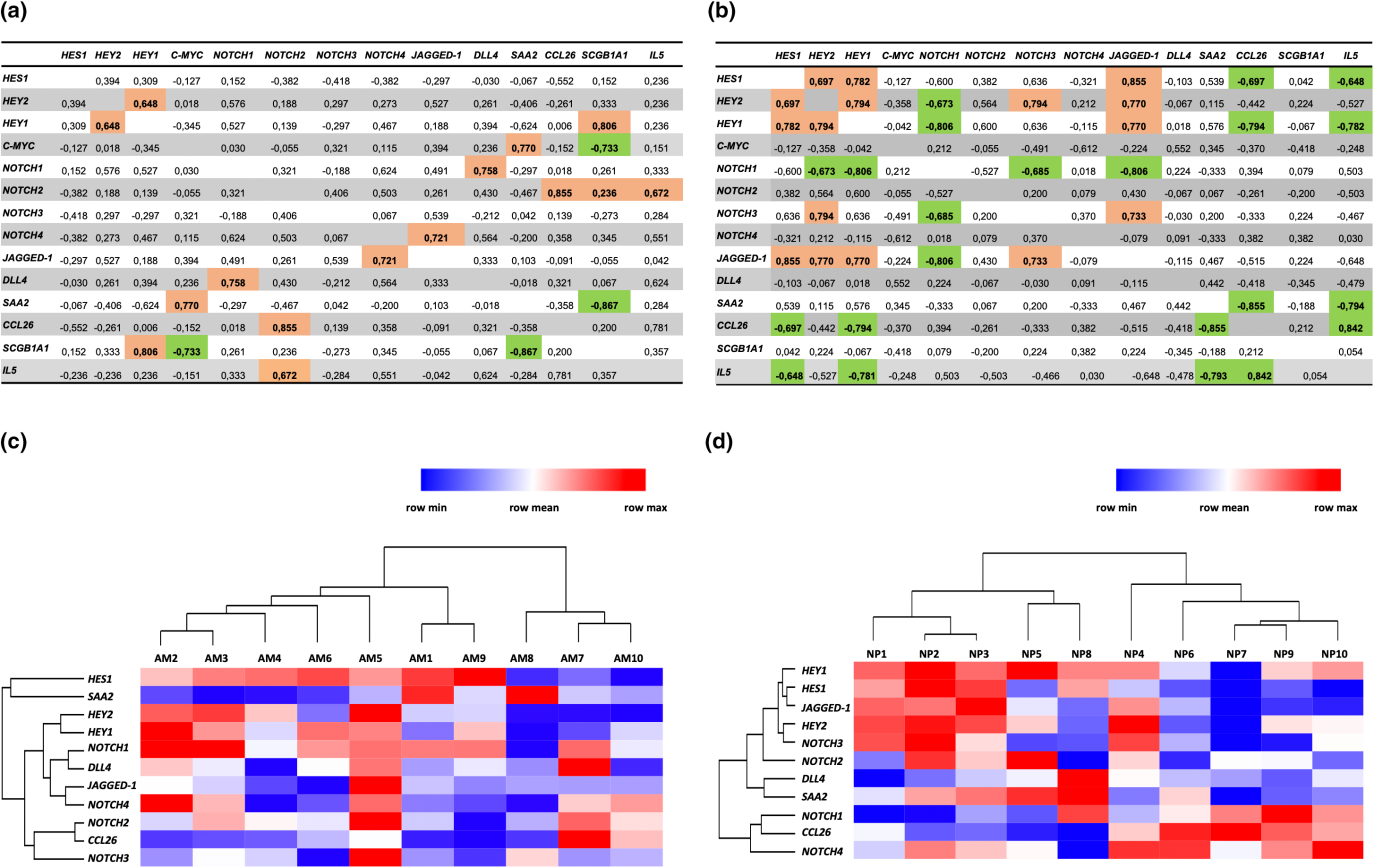
Spearman's correlation analysis of the expression levels of the indicated mRNAs analyzed in nasal polyps and adjacent mucosa biopsies. Spearman's rank is reported for each correlation in AM (a) and NP (b) biopsies, respectively. Values in bold are different from 0 with a significance level alpha = 0.05. Orange and green boxes highlight positive and negative correlation, respectively. Heat map of hierarchical clustering of gene expression in AM (c) and NP (d) tissues from each patient. The columns indicate patient number, and the rows indicate the analyzed genes. Upregulated genes are shown in red and downregulated genes are shown in blue. The intensity of color is proportional to the relative mRNA levels of transcription.

To compare the Notch‐related gene expression signature in AM and NP biopsies, we performed correlation analyses using the ΔCt values, a measure of the expression levels of each mRNAs. In the AM group, *DLL4* correlated positively with *NOTCH1* (*r* = 0.758; *p* = 0.015) and *HEY1* correlated positively with *HEY2* (*r* = 0.648; *p* = 0.049). A positive correlation was found between *NOTCH4* and *JAGGED‐1* (*r* = 0.721; *p* = 0.023). Lastly, we found that *SCGB1A1* correlated positively with *HEY1* (*r* = 0.806; *p* = 0.007). No other statistically significant correlation was detected (Figure 3a and Supplementary Table S2). In NPs, *JAGGED‐1* correlated positively with *NOTCH3* (*r* = 0.733; *p* = 0.020), *HEY1* (*r* = 0.770; *p* = 0.013), *HEY2* (*r* = 0.770; *p* = 0.013) and *HES1* (*r* = 0.855; *p* = 0.003). Moreover, *NOTCH3* correlated negatively with *NOTCH1* (*r* = −0.685; *p* = 0.035), and the latter correlated negatively with *JAGGED‐1* (*r* = −0.806; *p* = 0.007), *HEY1* (*r* = −0.806; *p* = 0.007) and *HEY2* (*r* = −0.673; *p* = 0.039). Furthermore, we found that *NOTCH3* correlated positively with *HEY2* (*r* = 0.794; *p* = 0.009; Figure 3b and Supplementary Table S3). This analysis suggests the existence in NPs, but not in AM, of a specific gene expression profile associated with Notch activation driven by the Notch ligand *JAGGED‐1*. In NP group, no association was found between the expression levels of any component of the Notch pathway and *C‐MYC* or *SCGB1A1*.

### 3.3 Stratification of NP and AM samples based on nasal mucosa gene expression profile

Hierarchical clustering analysis was performed to identify clusters of Notch pathways genes in NP and their relationship to the content of eosinophils. As shown in the heat map in Figure.3c, in AM there were no evident sample clusters associated with genes belonging to the Notch signaling pathway, reflecting the results obtained by correlation analysis (Figure 3a). Conversely, in NP (Figure 3d), this analysis showed the existence of two sample clusters: one cluster characterized by lower expression of *CCL26* and high expression of *SAA2* (putative non‐eosinophils, NEC‐NPs) and a second cluster characterized by higher expression of *CCL26*, and low expression of *SAA2* (putative eosinophils, EC‐NPs). In the first cluster, three of the five NP samples (NP 1, 2, and 3) displayed a relative high expression of *NOTCH3, JAGGED‐1, HEY1, HEY2*, and *HES1*. On the contrary, four of the five NP samples in the second cluster (NP 6, 7, 9, and 10) displayed a relative low expression of *NOTCH3, JAGGED‐1, HEY2*, and *HES1*. The remaining three NP samples showed a mixed Notch expression phenotype.

## 4 DISCUSSION

Nasal polyps are focal chronic flogistic extroflexions of sinonasal mucosa that progressively increase in number and size and occlude the nasal cavities, constituting an obstacle to the correct sinus breathing and drainage. Another feature of this disease is the tendency to recur and, in most cases, the association of medical and surgical therapy is necessary to manage this pathology (Nordin et al., 2013). Polyposis is a multifactorial disease with a complex pathophysiology involving multiple molecular mechanisms activated by infiltration, activation, and release of mediators by eosinophils, mast cells, and basophils (Schleimer, 2017). Due to the presence of inflammation mediators, cell proliferation is increased in the epithelium of NPs (Hsu et al., 2002). Despite being one of the most frequent sinonasal diseases (Rajguru, 2014), the etiology of polyposis is not yet clarified (Mahdavinia & Grammer 3rd, 2013) and this hampers the possibility of implementing targeted prevention treatments (Avdeeva & Fokkens, 2018). In this study, we found different mRNA levels of Notch receptors (*NOTCH1*, *2*, *3*, and *4*), ligands (*JAGGED‐1* and *DLL4*), and the target genes (*HES1*, *HEY1*, and *HEY2*) in NP, compared to AM. The picture that emerged was quite complex, but the differences observed between NPs and matched AM suggest a role for Notch in NPs formation. In NPs, but not in AM, we found a positive correlation among the expression levels of *NOTCH3*, *JAGGED‐1*, and Notch target genes *HEY1*, *HEY2*, and *HES1*, suggesting that the activation of the Notch pathway in NPs could be dependent by the ligand *JAGGED‐1*, and not by *DLL4*. Our data are consistent with recent work showing that *JAGGED‐1* elicits Th2 responses, thus stimulating the airway inflammation (Tindemans et al., 2017), and in nasal polyps epithelial cells the *JAGGED‐1* mediated activation of Notch signaling increases nuclear and cytoplasmic levels of *IL‐33 (*Chiappara et al., 2019 *)*. Along with Notch components, we also measured the expression levels of two relevant genes in NPs formation which are also Notch target: *C‐MYC* and *SCGB1A1*. We found that 50% of our NPs patients presented an elevated expression of *C‐MYC*, compared to their AM, but there was no correlation between *C‐MYC* and any of the Notch components, suggesting that in our NP samples *C‐MYC* is not regulated uniquely by Notch. *SCGB1A1* resulted also upregulated in 60% of NPs of CRS patients compared to their AM. Recently, it has been shown that *SCGB1A1* expression is decreased in both CRSsNP and CRSwNP and protein levels inversely correlated with the number of total infiltrating cells and symptom scores (Liu et al., 2009; Lu et al., 2011). Sssimilar to *C‐MYC*, in NP samples, we did not find any correlation between *SCGB1A1* with any Notch component, suggesting that, as for *C‐MYC*, in the study population, *SCGB1A1* is not under the exclusive control of Notch. In NPs with lower expression of *CCL26* and mostly high expression of *SAA2* (putative NE‐NPs) we found higher levels of Notch signaling, whereas in NPs with lower expression of *SAA2* and mostly high expression of *CCL26* (putative EC‐NPs) the molecular data were indicative of a lower Notch signaling. This observation could explain why EC‐NPs are more sensitive to glucocorticoids (GC) treatment compared to NEC‐NP (Wen et al., 2012), since there is evidence suggesting the involvement of the Notch pathway in resistance to treatment with GC. In vivo and in vitro experiments show that the silencing of *HES1* is required for proper GC signaling and overexpression of *HES1* leads to GC resistance, while *HES1* knockdown increases sensitivity to GC, by upregulating the expression level of the GC receptor (Revollo et al., 2013). Of interest, *HES1* was downregulated, compared to AM, in the majority of NPs analyzed in our study, which were all removed from patients that underwent GCs treatment. It should be considered that GCs treatment in these patients could have altered the transcription profiles of the NP, including the expression profile of the Notch signaling. It is indeed known that GCs influence Notch signaling in some cells and tissues. In asthmatic mouse model, GC inhibits Notch and modulates Th1 and Th2 responses thus ameliorating the airway inflammation (Hu et al., 2018). Additionally, in osteoblasts transfected with Dll1 or active Notch1 cortisol opposes the transcription of Hey1, Hey2, and HeyL genes (Zanotti et al., 2018). Furthermore, in MC3T3 cells, an osteoblast cell line, cortisol caused a time‐dependent increase in Notch1 and 2 mRNA levels, whereas Notch3 and 4 were not detected in the presence or absence of cortisol. MC3T3 cells expressed Dll1 and Jagged1 but not Jagged2 or Dll3 mRNAs, and cortisol did not have a substantial effect on the expression of any of these ligands (Pereira et al., 2002). These experiments show that the effects of GC are context‐dependent and are determined by the number and type of Notch receptors/ ligands expressed in a specific cell type and, thus, further investigation in vitro and in vivo are needed to characterize the effects of GC on Notch signaling in NP. The characterization of the crosstalk in NPs could open new therapeutic option for NP by combining GCs with Notch inhibitors (Piggott et al., 2011).

## 5 CONCLUSION

In conclusion, we report a specific *JAGGED‐1*/*NOTCH3*‐related gene expression profile in NPs of CRS patients suggesting a role for Notch signaling in the pathophysiology of polyposis. Additionally, we found higher levels of Notch signaling in NP with lower eosinophils infiltration. Hence, it could be interesting to evaluate the effect of Notch inhibitors, in combination with GC, as a possible therapeutic treatment for these NPs which are less sensitive to GC treatment.

This study has several limitations: (1) the small number of samples tested, (2) the choice of the adjacent mucosa turned out to be not a suitable control, (3) the molecular analyses based on mRNA determination that will have to be confirmed on a protein level, and (4) the putative EC‐NPs and NEC‐NP definition, based on mRNA determination, which will have to be confirmed by histological analyses. Nevertheless, we believe that our findings could be useful and guide other studies on the role of Notch in nasal polyps by providing information (1) on the choice of the most suitable control tissue, and (2) on which components of the pathways are expressed in nasal polyps. Specifically, on the choice of control, since there are not many data on the expression levels of the components of the Notch pathways in normal airways mucosa, we did not know what to expect in terms of interindividual variability relative to Notch, thus we reasoned that the best control would be the normal tissue adjacent to the polyp (as described by Fritz et al., 2003). Nevertheless, our results, in agreement with Zhu et al. (2018), showed that some characteristic of the polyps were present in the adjacent normal tissue, suggesting infiltration of inflammatory elements. Based on these findings, future studies will have to include normal tissue from a region further apart from the polyp. Additionally, our results could guide other studies on how to obtain maximum information from a small specimen. Based on our data, if available tissue is limited, the best approach could be to focus on the panel of most relevant Notch pathway components in NP (*NOTCH3, JAGGED‐1 HEY1, HEY2*, and *HES1*) and investigate eosinophils contents by immunohistochemistry. Therefore, this study has to be considered a pilot study aimed to identify the best experimental conditions to investigate the involvement of Notch in the pathophysiology of NPs and to establish if Notch could represent a novel therapeutic target or biomarker for disease progression or response to treatment in CRSsNP patients.

### AUTHOR'S CONTRIBUTIONS

A.A., C.Z., and S.P. participated in the recruitment of patients. G.A. and L.M. were responsible for the conception and design of the experiments and for the writing of the manuscript. V.M. and F.C. implemented the experiments, F.V.D.S., F.F., and P.A. participated in the evaluation and data analysis. P.R. and N.M. contributed to the writing of the manuscript and approved the submitted version. All authors have read and agreed to the published version of the manuscript.

## FUNDING INFORMATION

The authors received no financial support for the research, authorship and/or publication of this article.

## CONFLICT OF INTEREST

The authors declare no conflict of interest.

## INFORMED CONSENT STATEMENT

Informed consent was obtained from all subjects involved in the study.

## Supporting information


Figure S1

Table S1

## Data Availability

The datasets used and/or analyzed during the current study are available from the corresponding author on reasonable request.
