# Supplementary material for: Characterization of the Notch pathway in nasal polyps of patients with chronic rhinosinusitis: A pilot study
Source: Physiol Rep. 2022 Aug 27;10(16):e15403. doi: 10.14814/phy2.15403 (PMC9419157; doi:10.14814/phy2.15403)
Supplement: Supplementary file 1 — Figure S1 [file PHY2-10-e15403-s002.docx]

**Supplementary Figures**


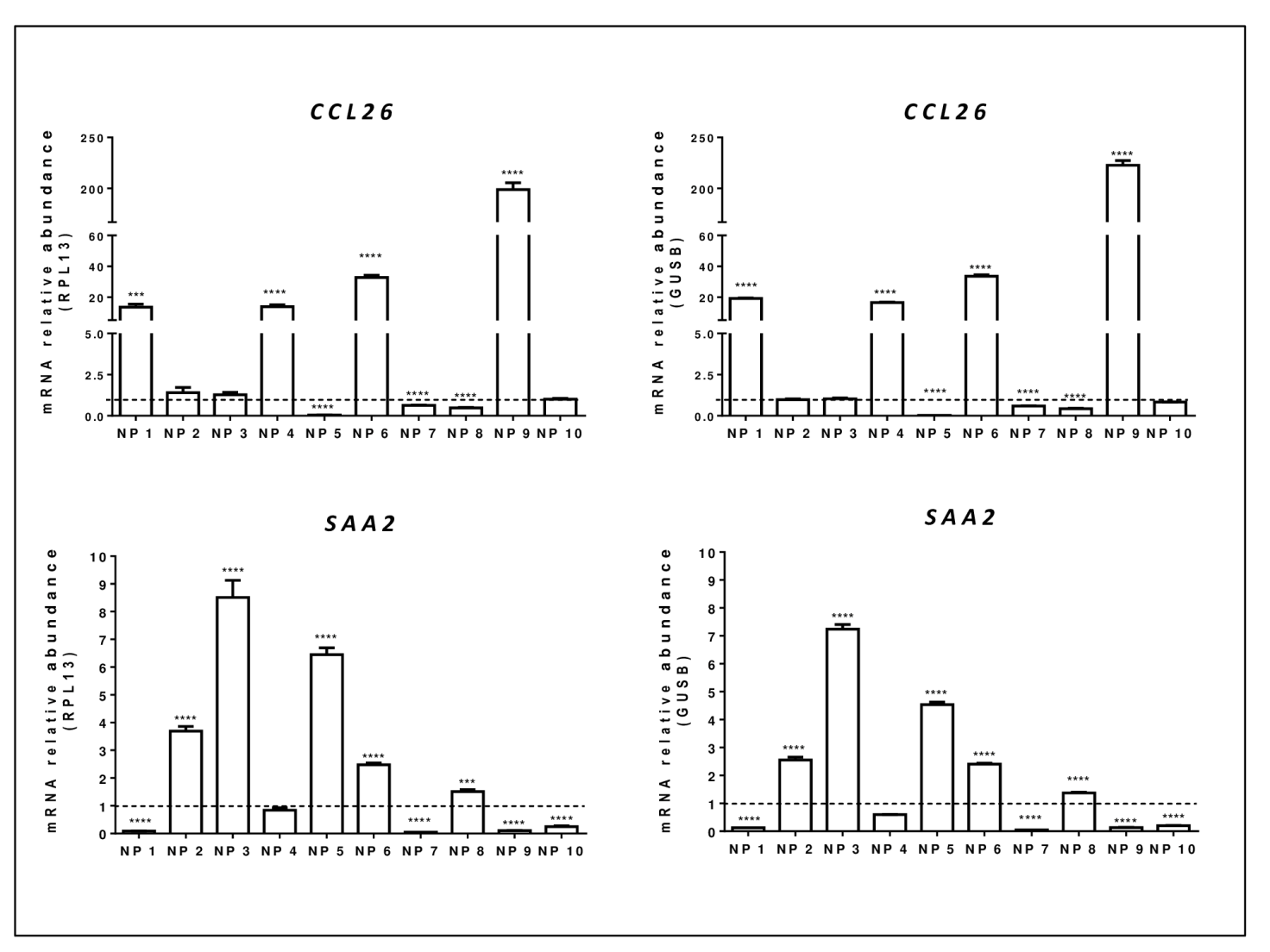


**Supplementary Figure 1. Expression of *CCL26* (c-c motif chemokine ligand 26) and *SAA2* (serum amyloid A2) genes in nasal polyps in patients with chronic rhinosinusitis.** Expression of *CCL26* and *SAA2* was assessed using qRT-PCR analysis. Relative changes in mRNA expression levels between nasal polyps (NP) and corresponding adjacent mucosa (AM) were calculated according to the 2^-ΔΔCt^ method using *RPL13A* or GUSB as reference gene. Results are expressed as mean ± SEM of at least three experiments. **** P < .0001, *** P < .001, ** P < .01 and * P < .05, NP vs the corresponding AM samples. The dotted line represents the normalized expression levels in AM samples.


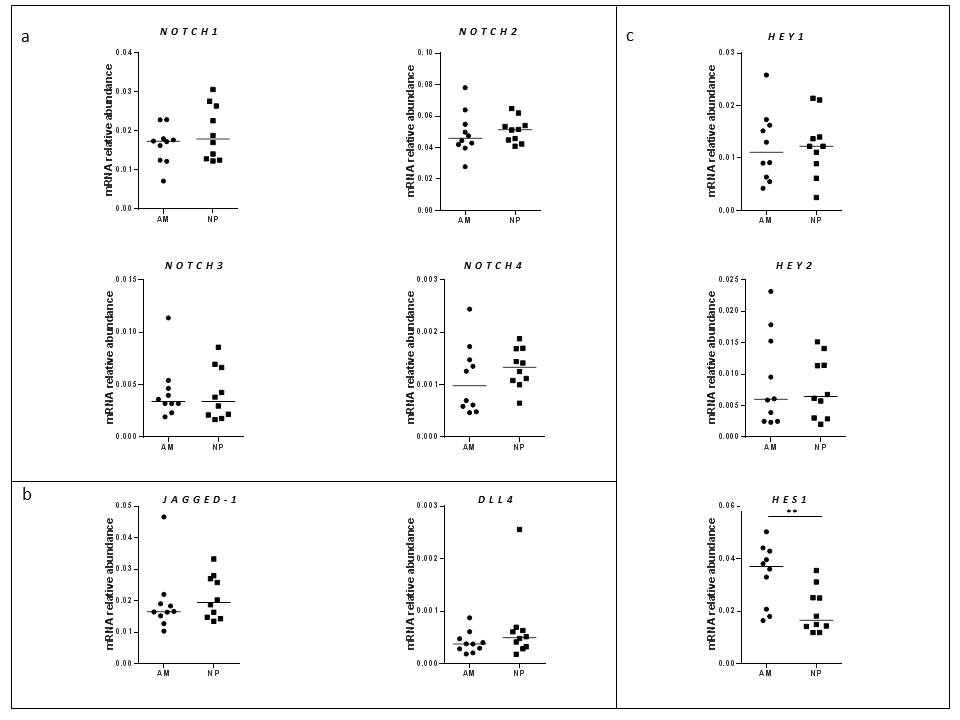


**Supplementary Figure 2.** **Expression of components of the Notch pathway in nasal polyps and adjacent mucosa in patients with chronic rhinosinusitis.** Gene expression of Notch receptors: a) *(NOTCH 1-4)*; b) ligands (*JAGGED-1, DLL4)*  and c) target genes (*HEY1, 2* and *HES1)* was measured using qRT-PCR. Delta Ct values (ΔCt = Ct target gene - Ct *RPL13*) were used to assess gene expression levels (2^-ΔCt^ are shown on the Y-axis). Results are shown as median ± SEM of at least three experiments. ** P < .01 NP vs AM samples. Comparison of gene expression levels, student t test.


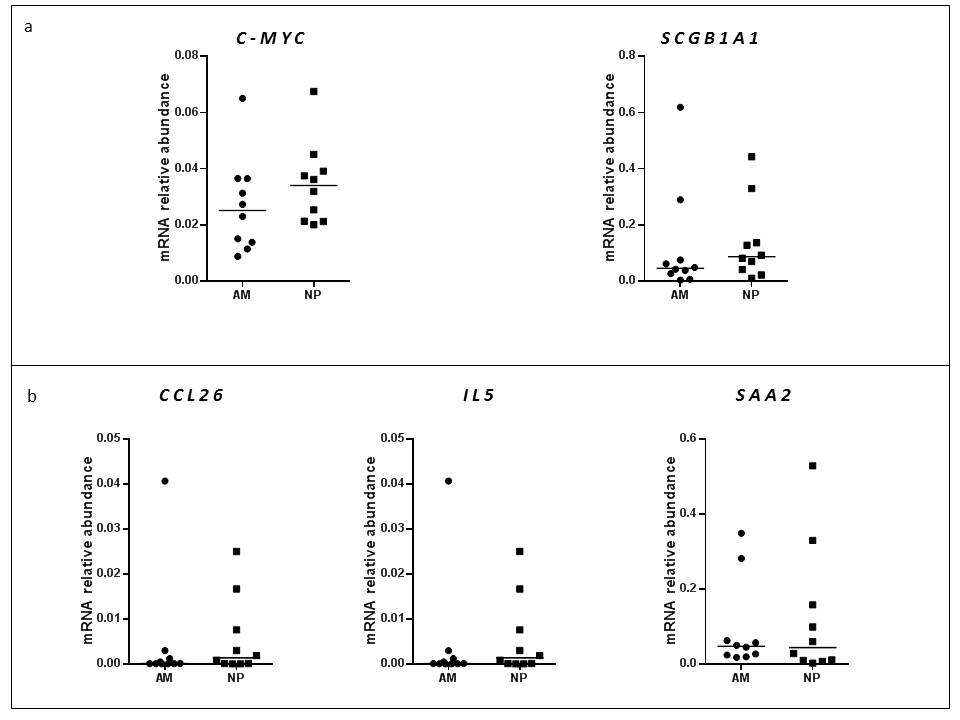


**Supplementary Figure 3. Expression of *C-MYC*, *SCGB1A1* (uteroglobin, member of the secretoglobin family), *CCL26* (c-c motif chemokine ligand 26), *IL5* (Interleukin-5) and *SAA2* (serum amyloid A2) in nasal polyps and adjacent mucosa in patients with chronic rhinosinusitis.** Expression of a) *C-MYC*, *SCGB1A1* and b) *CCL26* , *IL5* and *SAA2* was assessed using qRT-PCR. Delta Ct values (ΔCt = Ct target gene - Ct *RPL13*) were used to assess gene expression levels (2^-ΔCt^ are shown on the Y-axis). Results are shown as median ± SEM of at least three experiments. Comparison of gene expression levels, student t test.
