# Supplementary material for: Characterization of the Notch pathway in nasal polyps of patients with chronic rhinosinusitis: A pilot study
Source: Physiol Rep. 2022 Aug 27;10(16):e15403. doi: 10.14814/phy2.15403 (PMC9419157; doi:10.14814/phy2.15403)
Supplement: Supplementary file 2 — Table S1 [file PHY2-10-e15403-s001.docx]

**Supplementary Tables**

**Supplementary Table 1:** Primers used for quantitative RT-PCR

|  | **HOMO SAPIENS PRIMER SEQUENCE (5’-3’)** |
| --- | --- |
| ***GUSB F*** | CCCGCGGTCGTGATGTGGTC |
| ***GUSB R*** | GCCGGGAGGGGTCCAAGGAT |
| ***RPL13A F*** | GGAGGTGCAGGTCCTGGTGCTT |
| ***RPL13A R*** | CGTACGACCACCACCTTCCGG |
| ***HEY1 F*** | CCGAGATCCTGCAGATGACCGT |
| ***HEY1 R*** | AACGCGCAACTTCTGCCAGG |
| ***HEY2 F*** | AAAAGGCGTCGGGATCG |
| ***HEY2 R*** | AGCTTTTTCTAACTTTGCAGATCC |
| ***HES1 F*** | CGGACATTCTGGAAATGACA |
| ***HES1 R*** | CATTGATCTGGGTCATGCAG |
| ***DLL4 F*** | GCGAGAAGAAAGTGGACAGG |
| ***DLL4 R*** | ATTCTCCAGGTCATGGCAAG |
| ***JAGGED-1 F*** | GACTCATCAGCCGTGTCTCA |
| ***JAGGED-1 R*** | TGGGGAACACTCACACTCAA |
| ***NOTCH1 F*** | GTCAACGCCGTAGATGACC |
| ***NOTCH1 R*** | TTGTTAGCCCCGTTCTTCAG |
| ***NOTCH2 F*** | CAGATGCGAGTGTGTCCCAGG |
| ***NOTCH2 R*** | TACCCCGAGTGCCTGGTGGGC |
| ***NOTCH3 F*** | GCCAAGCGGCTAAAGGTAGA |
| ***NOTCH3 R*** | TGAGTCCACTGACGGCAATC |
| ***NOTCH4 F*** | CAACTGCCTCTGTCCTGATG |
| ***NOTCH4 R*** | GCTCTGCCTCACACTCTG |
| ***C-MYC F*** | CGACTCTGAGGAGGAACAAG |
| ***C-MYC R*** | TGCGTAGTTGTGCTGATGTG |
| ***CCL26 F*** | CTGTGATATTCACTACCAAAAGAGG |
| ***CCL26 R*** | CCAAGCGTCCTCGGATGAAA |
| ***SCGB1A1 F*** | AAGCATCATTAAGCTCATGGAAAA |
| ***SCGB1A1R*** | GTGGACTCAAAGCATGGCAG |
| ***SAA2 F*** | ATACTTCCATGCTCGGGGGA |
| ***SAA2 R*** | TGGATATTCTCTCTGGCATTGCTGA |
| ***IL5 F*** | TGCTGATAGCCAATGAGACTCTG |
| ***IL5 R*** | ACCCCCTTGCACAGTTTGAC |

**Supplementary Table 2:** p values associated to the results of correlations analyses among the expression levels of mRNAs analyzed in AM samples

|  | ***HES1*** | ***HEY2*** | ***HEY1*** | ***C-MYC*** | ***NOTCH1*** | ***NOTCH2*** | ***NOTCH3*** | ***NOTCH4*** | ***JAGGED-1*** | ***DLL4*** | ***SAA2*** | ***CCL26*** | ***SCGB1A1*** | ***IL5*** |
| --- | --- | --- | --- | --- | --- | --- | --- | --- | --- | --- | --- | --- | --- | --- |
| ***HES1*** |  | 0,263 | 0,387 | 0,733 | 0,682 | 0,279 | 0,233 | 0,279 | 0,407 | 0,946 | 0,865 | 0,105 | 0,682 | 0,513 |
| ***HEY2*** | 0,263 |  | **0,049^a^** | 0,973 | 0,088 | 0,607 | 0,407 | 0,448 | 0,123 | 0,470 | 0,247 | 0,470 | 0,349 | 0,513 |
| ***HEY1*** | 0,387 | **0,049** |  | 0,330 | 0,123 | 0,707 | 0,407 | 0,179 | 0,607 | 0,263 | 0,060 | 1,000 | **0,007** | 0,513 |
| ***C-MYC*** | 0,733 | 0,973 | 0,330 |  | 0,946 | 0,892 | 0,368 | 0,759 | 0,263 | 0,513 | **0,013** | 0,682 | **0,020** | 0,682 |
| ***NOTCH1*** | 0,682 | 0,088 | 0,123 | 0,946 |  | 0,368 | 0,607 | 0,060 | 0,155 | **0,015** | 0,407 | 0,973 | 0,470 | 0,349 |
| ***NOTCH2*** | 0,279 | 0,607 | 0,707 | 0,892 | 0,368 |  | 0,247 | 0,144 | 0,470 | 0,218 | 0,179 | **0,003** | 0,513 | **0,039** |
| ***NOTCH3*** | 0,233 | 0,407 | 0,407 | 0,368 | 0,607 | 0,247 |  | 0,865 | 0,114 | 0,560 | 0,918 | 0,707 | 0,448 | 0,427 |
| ***NOTCH4*** | 0,279 | 0,448 | 0,179 | 0,759 | 0,060 | 0,144 | 0,865 |  | **0,023** | 0,096 | 0,584 | 0,313 | 0,330 | 0,105 |
| ***JAGGED-1*** | 0,407 | 0,123 | 0,607 | 0,263 | 0,155 | 0,470 | 0,114 | **0,023** |  | 0,349 | 0,785 | 0,811 | 0,892 | 0,918 |
| ***DLL4*** | 0,946 | 0,470 | 0,263 | 0,513 | **0,015** | 0,218 | 0,560 | 0,096 | 0,349 |  | 0,973 | 0,368 | 0,865 | 0,06 |
| ***SAA2*** | 0,865 | 0,247 | 0,060 | **0,013** | 0,407 | 0,179 | 0,918 | 0,584 | 0,785 | 0,973 |  | 0,313 | **0,002** | 0,427 |
| ***CCL26*** | 0,105 | 0,470 | 1,000 | 0,682 | 0,973 | **0,003** | 0,707 | 0,313 | 0,811 | 0,368 | 0,313 |  | 0,584 | 0,011 |
| ***SCGB1A1*** | 0,682 | 0,349 | **0,007** | **0,020** | 0,470 | 0,513 | 0,448 | 0,330 | 0,892 | 0,865 | **0,002** | 0,584 |  | 0,313 |
| ***IL5*** | 0,513 | 0,513 | 0,513 | 0,682 | 0,349 | **0,039** | 0,427 | 0,105 | 0,918 | 0,060 | 0,427 | 0,011 | 0,313 |  |

^a^  Bold characters highlight significant p values (< 0.05)

**Supplementary Table 3:** p values associated to the results of correlations analyses among the expression levels of mRNAs analyzed in NP samples

|  | ***HES1*** | ***HEY2*** | ***HEY1*** | ***C-MYC*** | ***NOTCH1*** | ***NOTCH2*** | ***NOTCH3*** | ***NOTCH4*** | ***JAGGED-1*** | ***DLL4*** | ***SAA2*** | ***CCL26*** | ***SCGB1A1*** | ***IL5*** |
| --- | --- | --- | --- | --- | --- | --- | --- | --- | --- | --- | --- | --- | --- | --- |
| ***HES1*** |  | **0,031^a^** | **0,011** | 0,733 | 0,073 | 0,279 | 0,054 | 0,368 | **0,003** | 0,785 | 0,114 | **0,031** | 0,918 | 0,049 |
| ***HEY2*** | **0,031** |  | **0,009** | 0,313 | **0,039** | 0,096 | **0,009** | 0,560 | **0,013** | 0,865 | 0,759 | 0,204 | 0,537 | 0,123 |
| ***HEY1*** | **0,011** | **0,009** |  | 0,918 | **0,007** | 0,073 | 0,054 | 0,759 | **0,013** | 0,973 | 0,088 | **0,009** | 0,865 | **0,011** |
| ***C-MYC*** | 0,733 | 0,313 | 0,918 |  | 0,560 | 0,892 | 0,155 | 0,067 | 0,537 | 0,105 | 0,330 | 0,296 | 0,233 | 0,492 |
| ***NOTCH1*** | 0,073 | **0,039** | **0,007** | 0,560 |  | 0,123 | **0,035** | 0,973 | **0,007** | 0,537 | 0,349 | 0,263 | 0,838 | 0,144 |
| ***NOTCH2*** | 0,279 | 0,096 | 0,073 | 0,892 | 0,123 |  | 0,584 | 0,838 | 0,218 | 0,865 | 0,865 | 0,470 | 0,584 | 0,144 |
| ***NOTCH3*** | 0,054 | **0,009** | 0,054 | 0,155 | **0,035** | 0,584 |  | 0,296 | **0,020** | 0,946 | 0,584 | 0,349 | 0,537 | 0,179 |
| ***NOTCH4*** | 0,368 | 0,560 | 0,759 | 0,067 | 0,973 | 0,838 | 0,296 |  | 0,838 | 0,811 | 0,349 | 0,279 | 0,279 | 0,946 |
| ***JAGGED-1*** | **0,003** | **0,013** | **0,013** | 0,537 | **0,007** | 0,218 | **0,020** | 0,838 |  | 0,759 | 0,179 | 0,133 | 0,537 | 0,049 |
| ***DLL4*** | 0,785 | 0,865 | 0,973 | 0,105 | 0,537 | 0,865 | 0,946 | 0,811 | 0,759 |  | 0,204 | 0,233 | 0,330 | 0,166 |
| ***SAA2*** | 0,114 | 0,759 | 0,088 | 0,330 | 0,349 | 0,865 | 0,584 | 0,349 | 0,179 | 0,204 |  | **0,003** | 0,607 | **0,009** |
| ***CCL26*** | **0,031** | 0,204 | **0,009** | 0,296 | 0,263 | 0,470 | 0,349 | 0,279 | 0,133 | 0,233 | **0,003** |  | 0,560 | **0,004** |
| ***SCGB1A1*** | 0,918 | 0,537 | 0,865 | 0,233 | 0,838 | 0,584 | 0,537 | 0,279 | 0,537 | 0,330 | 0,607 | 0,560 |  | 0,892 |
| ***IL5*** | 0,049 | 0,123 | **0,011** | 0,492 | 0,144 | 0,144 | 0,179 | 0,946 | 0,049 | 0,166 | **0,009** | **0,004** | 0,892 |  |

^a^ Bold characters highlight significant p values (< 0.05)
